# Identification of Haptoglobin as a Potential Biomarker in Young Adults with Acute Myocardial Infarction by Proteomic Analysis

**DOI:** 10.21315/mjms2020.27.2.8

**Published:** 2020-04-30

**Authors:** Norbaiyah Mohamed Bakrim, Aida Nur Sharini Mohd Shah, Norlelawati A Talib, Jamalludin Ab Rahman, Aszrin Abdullah

**Affiliations:** 1 Department of Basic Medical Sciences, Kulliyyah of Medicine, International Islamic University Malaysia, Pahang, Malaysia; 2 Department of Internal Medicine, Kulliyyah of Medicine, International Islamic University Malaysia, Pahang, Malaysia; 3 Department of Pathology and Laboratory Medicine, Kulliyyah of Medicine, International Islamic University Malaysia, Pahang, Malaysia; 4 Department of Community Medicine, Kulliyyah of Medicine, International Islamic University Malaysia, Pahang, Malaysia

**Keywords:** myocardial infarction, young adults, plasma, proteins, electrophoresis

## Abstract

**Background:**

Acute myocardial infarction (AMI) molecular research in young adults is still limited. The aim of this study is to identify AMI proteomic biomarker(s) in young adults.

**Methods:**

This study comprised of two phases namely discovery and verification. In the discovery phase, proteins in the pooled plasma samples from young male adults between 18 and 45 years (10 AMI patients and 10 controls) were separated using two-dimensional electrophoresis. The protein spots that were expressed differently in the AMI patients were identified via matrix-assisted laser desorption/ionisation time-of-flight mass spectrometry. The plasma concentrations of these proteins were quantified using enzyme-linked immunosorbent assay during the verification phase (40 AMI patients and 80 controls).

**Results:**

Haptoglobin (Hp), apolipoprotein AI (Apo AI) and apolipoprotein AIV (Apo AIV) were up-regulated in the discovery phase. In the verification phase, the plasma concentration of Hp was significantly higher in AMI patients than the controls (*P* < 0.001). Logistic regression showed an association between Hp and AMI in young adults (odds ratio [OR] = 1.016, 95% CI: 1.002–1.030, *P* = 0.025) independent of other AMI risk factors. Hp was significantly correlated with high sensitivity C-reactive protein (hs-CRP) (*r* = 0.424, *P* < 0.001).

**Conclusion:**

In young adults with AMI, plasma Hp concentrations were elevated and it is independently associated with AMI. A positive correlation with hs-CRP suggests Hp could be a potential biomarker of AMI in young adults.

## Introduction

Acute myocardial infarction (AMI) is the leading cause of premature deaths worldwide. Despite an overall gradual decline in AMI-related hospitalisations over the past few decades, the occurrence of AMI in young adults is increasing (1–3). The prevalence of young adults with AMI varies across populations with higher percentage was noted in the developing countries, particularly in the South Asian region compared to the developed countries (4–7). As for Malaysia, 16.0% of AMI admissions were young patients defined as less than 45 years old for male and 55 years old for female (8). Evidently, the definition of ‘young’ AMI differs across studies, with the age range being 35–55 years old (9–14). Nevertheless, the most frequently-employed cut-off age is 45 years old (8, 13, 15–17). Unfortunately, young AMI commonly manifests in males, who are usually the main breadwinners of families (18). Thus, the diagnosis of AMI at this prime age exerts more significant health, socioeconomic and psychological toll on not only the patients but their families and the community as well.

Given the significant health and economic burden of young AMI, the discovery of biomarker specific to this age group is essential to improve the risk stratification, diagnosis and prognostication of the disease. Additionally, such discoveries may help unearth the reason behind the accelerated atherosclerotic process, which leads to the early emergence of AMI in young adults. Apart from the studies on risk factor profiles, clinical presentations and outcome of young adults with AMI, research into the specific molecular characterisations of young AMI remain limited. Previous studies have reported the distinct features between young and elderly AMI patients. Smoking has been identified to be the most important risk factor for young AMI, while dyslipidemia, hypertension and diabetes were recognised to play more significant roles in the disease among the elderly (19–20). Meanwhile, single vessel diseases were more frequent among young adults with AMI compared to their older counterparts (21). Therefore, it is hypothesised that beyond the discrepancies in the risk factor profiles and clinical presentations, young adults with AMI might be associated with difference pathophysiological changes that are reflected by the changes in the protein expression profiles.

Evidently, proteomic analyses have brought to light the modifications in the protein-expression profiles of diseased tissues in comparison to the control. These, in turn, have facilitated the discovery of the potential biomarkers. Mateos-Cáceres et al. (22) conducted a plasma proteomic study on acute coronary syndrome (ACS) patients and identified four modified proteins, namely alpha-1 antitrypsin, apolipoprotein AI (Apo AI), fibrinogen (gamma chain) and immunoglobin gamma (heavy chain). Meanwhile, in a review article, 16 other potential proteomic markers for AMI with different functions in the disease development were identified (23). However, only several biomarkers have been validated following their identification in the discovery phase. Recently, a three-phased (discovery, verification and validation) proteomic analyses by Basak et al. (24) have discovered that the down-regulation of apolipoproteins and albumin might be responsible for the impairment of the reverse cholesterol pathway during atherosclerosis in coronary artery disease (CAD) patients.

It is important to note that few studies on plasmic proteomes of AMI have analysed samples from elderly patients since the disease was more prevalent in this age group. In fact, elderly AMI patients usually present with other comorbidities that require pharmacological interventions, which may affect the protein profiles (25). Hence, the identified proteomic biomarkers might not accurately reflect the protein expression profile modifications in the younger population. Therefore, the identification of potential proteomic biomarkers that are specific to young AMI patients constitutes the preliminary step to the formulation of personalised management strategies, both for disease prevention and therapeutic interventions. The main objective of this study is to compare plasma proteomic profiles between young AMI patients and controls with the aim to determine the potential role of Hp as a biomarker for AMI in young adults.

## Methods

### Study Design, Sample Size Calculation and Subject Recruitment

This is a case-control study on male AMI patients of age 18–45 years old who were newly-diagnosed with ST elevated myocardial infarction (STEMI) or non-ST elevated myocardial infarction (NSTEMI) at the Emergency Department of Hospital Tengku Ampuan Afzan (HTAA), Kuantan, Pahang, Malaysia. The diagnosis of AMI was made in view of the presence of prolonged chest pain, typical changes in a 12-lead electrocardiogram (ECG), and/or elevated serum creatine kinase (CK) levels. The control subjects consisted of healthy male volunteers between 18 and 45 years old who were recruited during health screening at selected outpatient clinics and higher education institutions. All subjects who were on long-term medications and/or had a history of chronic illnesses such as diabetes, neoplasms, infections and autoimmune diseases were excluded from the study to minimise the confounding factors of the proteomic profiling.

For the discovery phase, the sample size was determined as described by Levin et al. (26) whereby the study defines the sample size for unknown protein in differential proteomic profiles analysis. Based on the assumption of the combination of the biological and technical variance of 75% between samples, the sample size of 10 in each group would be adequate to detect changes (up or down regulation) in a given protein with at least 80% power and a *P*-value of 0.05. The subjects selected were matched for age, all current smokers, and body mass index (BMI) in the range between 15.00 kg/m^2^ and 30.00 kg/m^2^ to minimise the confounding effects on the protein expression profiles.

The discovery phase is followed by a verification phase to allow the expression of proteins identified in the first phase to be measured in the individual sample with larger sample size. For the verification phase, the sample size was calculated using OpenEpi software. The sample size of 40 for AMI patients and 80 for controls were able to detect differences between the means with 0.05 significant levels at a power of 95% (27). In this study, the total number of participants in the controls was doubled (1:2 ratio) to increase the power of the sample size.

### Socio-Demographic and AMI Risk Factor Profiles

Socio-demographic data and AMI risk factor profiles were assessed in all subjects. Smoking pack-years was calculated by multiplying the number of packs of cigarettes smoked per day by the number of years the person has smoked. BMI was calculated by the weight of a subject in kilograms divided by his height in meters squared (kg/m^2^). Blood pressure was measured twice in 10 min apart on the day of recruitment and the average of two readings was calculated.

### Blood Sample Collection

About 10 mL of blood was collected from AMI patients in the Emergency Department of HTAA prior to the administration of thrombolysis or percutaneous coronary intervention (PCI) procedure to ensure the proteomic profiles accurately reflect the recent AMI instead of other invasive interventions. The blood was transferred into an ethylenediaminetetraacetic acid (EDTA) blood tube and centrifuged at 2,500 revolutions per min (rpm) for 10 min to separate the plasma and the plasma was stored in a −80 °C freezer pending further proteomic works. Data for biochemical profiles such as fasting glucose, total cholesterol and high sensitive C-reactive protein (hs-CRP) were obtained from the patient’s case note during admission. As for the control subjects, they were requested to fast overnight prior to blood taking procedure and 20 mL of blood was collected on the following day. Half of the blood was transferred into an EDTA tube for proteomic analysis while another 10 mL of blood was transferred into a sodium citrate tube to be analysed for biochemical profiles.

### Discovery Phase

The plasma of 10 individuals within each group was pooled and prepared in triplicates. Albumin was removed using ProteoExtract Albumin Removal Kit (Merck, Darmstadt, Germany). A total of 500 μg of protein was precipitated using ReadyPrep 2-D Cleanup Kit (Bio-Rad) as per the manufacturer’s instructions. The sample volume of 125 μL equal to 169 μg of protein was loaded onto IPG strips (Bio-Rad, ReadyStrip pH 4–7) with a length of 7 cm and was allowed to passively rehydrate for 12 h. Then, the first dimension of protein separation was performed by means of isoelectric focusing at a maximum current of 50 μA per strip at 20 °C, in accordance with the user manual of the PROTEAN IEF Cell System (Bio-Rad). As for the second dimension, the strip was carefully placed on a 12% sodium dodecyl sulphate (SDS) gel with 0.5% Agarose. Vertical 2-dimensional electrophoresis (2-DE) was performed at 120 V until the blue dye of the Agarose ended up at the opposite end of the gel. The gel was stained with Coomassie Brilliant Blue R-250 and later de-stained until the background was acceptable. The gels were then scanned using UMAX POWERLOOK 1000 (UMAX Technologies, Inc), and the images were analysed using PD Quest 7.2.0 2-D analysis software (Bio-Rad). The intensities of the protein spots in the gels were assumed to be proportional to the quantity of the protein present.

### Protein Identification

The protein spots that were differentially expressed between the two groups were manually excised from the gel and digested by trypsin. These proteins of interest were analysed via matrix-assisted laser desorption/ionisation time of flight (MALDI-TOF) 5800 (AB Sciex) mass spectrometer. Laser ultraviolet was directed towards the sample molecules and matrix. The laser energy was converted into molecular electronic energy for desorption and ionisation. Each peptide was fragmented within the mass spectrometer to produce ions that provided amino acids sequence information. The peptide sequences were identified by means of the determination of the masses of the intact peptides, thereby generating a ‘peptide mass fingerprint’ for each protein spot. The Mascot (Matrix Science) sequence-matching software was utilised to identify the proteins by comparing the ‘peptide mass fingerprints’ against the Homo sapiens MSPnr 100 database.

### Verification Phase

The plasma concentrations of the proteins identified during the discovery phase were quantified using enzyme-linked immunosorbent assays (ELISA) (Elabscience®, USA). The standard working solution or sample (100 μL) was pipetted into the antibody pre-coated ELISA microplate wells. The plate was incubated at 37 °C for 90 min. The protein-antibody complex was detected by a biotinylated detection antibody specific to the targeted protein and successively was recognised by Avidin-Horseradish Peroxidase (HRP) conjugate. Substrate reagent (90 μL) was pipetted into each well to bind to the protein-biotinylated detection antibody-HRP binding. This enzyme-substrate reaction was ceased by the addition of 50 μL Stop Solution and the reaction was measured in optical density unit by a microplate reader (Infinite 200 PRO, TECAN, Switzerland) at 450 nm wavelength. The concentrations of the proteins were calculated with respect to the equation of standard graph, whereby the *x*- and *y-*axes denoted the standard concentrations and optical density (OD) values, respectively.

### Statistical Analysis

The baseline characteristics of the subjects and the plasma concentrations of the proteins were presented as means (standard deviations [SD]) and median (interquartile range [IQR]) as indicated. Comparison of data in cases and controls were analysed using the independent Student’s *t*-test for normally distributed data and Mann Whitney U test for not normally distributed data. The association between the newly-identified protein biomarker(s) and AMI were evaluated using multivariable binary logistic regressions, which were adjusted for other known risk factors of AMI such as age, smoking status (yes/no), total cholesterol, fasting blood glucose and blood pressure. The results were presented in terms of adjusted odds ratio (AOR), 95% confidence intervals (CI) and *P*-values. The correlation between the newly identified potential biomarker and hs-CRP was determined using Spearman’s test as the hs-CRP was non-normally distributed. All statistical analyses were performed using IBM Statistical Package for Social Sciences (SPSS) 24.0 software and *P*-value < 0.05 was considered as significant.

## Results

### Baseline Characteristics of Study Subjects

Table 1 shows the baseline characteristics of all subjects for the discovery and verification phases. All numerical variables were normally distributed except for smoking pack-years and fasting blood glucose. In the discovery phase, subjects for case and control were matched for age with no significant inter-group differences in BMI, total cholesterol levels and systolic blood pressures (SBP). The subjects were more heterogeneous in the verification phase, whereby the risk factors of AMI such as age, BMI, prevalence of smoking, smoking pack-years, blood pressure and fasting blood glucose levels were significantly greater in the AMI patients compared to the controls.

### Two-Dimensional Protein Electrophoresis

**Figure 1:**
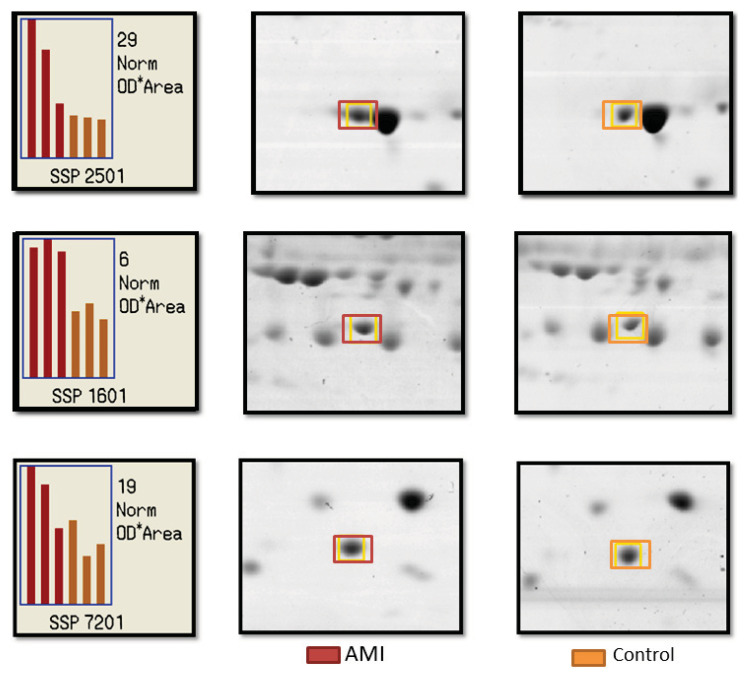
Representative patterns of protein spots that were differently expressed in young AMI patients as compared to the controls, recognised as SSP 2502, SSP 1601 and SSP 7201. The spots in the 2-dimentional electrophoresis gels of pH 4–7 were examined and analysed using the PD Quest 7.2.0 software. The graph corresponds with the intensity of the protein in three gels for each group in terms of optical density (OD). Note: Red bars denote data from the AMI group while orange bars denote data from control group

More than 100 protein spots were observed in each of the 2-DE protein map gel. The coefficients of correlation of the three sample gels in the same group were more than 0.8, hence denoting the good consistency of the experimental system. Three protein spots (SSP 7201, SSP 2501 and SSP 1601) were significantly expressed in the AMI patients compared to the controls (*P* < 0.05) as shown in Figure 1. Following MALDI-TOF analysis, the three spots of interest were identified as haptoglobin (Hp) (SSP 7201), apolipoprotein AI (Apo AI) (SSP 2501) and apolipoprotein AIV (Apo AIV) (SSP 1601) as demonstrated in Table 2.

### ELISA Analysis

**Figure 2:**
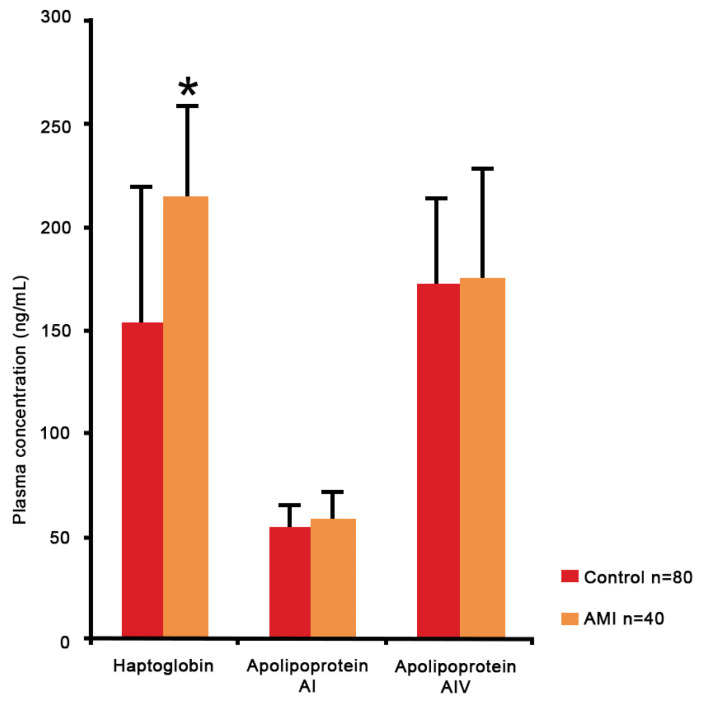
Plasma concentrations of Hp, Apo AI and Apo AIV during verification phase in 40 young AMI patients and 80 controls. Notes: Data are presented as means (SD); *Hp was expressed significantly difference (*P* < 0.001) between the groups as per the outcomes of the independent Student’s *t*-test

Data for plasma concentrations of Hp, Apo AI and Apo AIV were normally distributed. Figure 2 demonstrates that the plasma concentration of Hp is significantly higher in the AMI patients than the controls; 214.71 (SD = 5.10) versus 153.38 (SD = 64.67) ng/mL, *P* < 0.001. Concurrently, the plasma concentrations of Apo AI and Apo AIV were also up-regulated, however there was no significant difference; 58.20 (SD = 15.82) versus 54.89 (SD = 14.88) ng/mL, *P* = 0.763 and 175.28 (SD = 53.85) versus 172.55 (SD = 42.68) ng/mL, *P* = 0.264, respectively.

Following adjustments for other known risk factors of AMI, logistic regression revealed that Hp concentration was significantly associated with young AMI (AOR = 1.016, 95% CI: 1.002–1.030, *P* = 0.025) as shown in Table 3. As concentrations of Hp were correlated with concentrations of hs-CRP using Spearman correlation, it showed a moderate correlation between both protein markers (*r* = 0.424, *P* < 0.001).

## Discussion

In this study, differential proteomic profiles analysis during discovery phase discovered that Hp, Apo AI and Apo AIV to be significantly expressed in young AMI patients relative to the healthy individuals. However, in a larger number of more heterogeneous samples in the verification phase, only plasma concentration of Hp was significantly higher in the AMI patients than the controls. Although the plasma concentrations of Apo AI and Apo AIV were also elevated in the AMI patients the increase was not significant.

The up-regulation of Hp in the AMI patients in this study was consistent with the results from a study by Lee et al. (27) as they observed a significantly higher plasma Hp concentration in coronary artery disease (CAD) patients vis-à-vis (versus) normal controls (262.4 [SD = 144.2] versus 176.0 [SD = 86.7] ng/mL, *P* < 0.001). The elevation of Hp in the AMI patients could have been in response to the acute inflammatory process subsequent to the recent cardiac injury. The peak concentration of hepatic mRNA of Hp occurred 24 h–48 h post-inflammation, after which it returned to baseline Hp expression levels within 2–7 days (28). The cytokine interleukin-6 (IL-6), which is the main mediator of the acute-phase inflammatory response, has been identified as the principal inducer of Hp gene expression (28). Hp is also known as an acute-phase reactant of non-cardiac origin that functions as a high-affinity haemoglobin-binding protein and an antioxidant (29). Evidently, the binding of Hp with highly-toxic free haemoglobin reduces oxidative tissue damage. Therefore, it has been proposed that high levels of Hp during AMI had a protective function as it was associated with a lower risk of heart failure within one year post-AMI (30). Consequently, Hp is likely to be a prognostic marker following AMI event owing to its antioxidant property.

Meanwhile, the up-regulation of Apo AI and Apo AIV in the discovery phase contradict several studies, which have found both proteins to be down-regulated in the AMI patients. For example, a study by Basak et al. (24) demonstrated a reduction in the plasma expression of Apo AI in stable CAD patients in both discovery and verification phases. Meanwhile, Kronenberg et al. (31) who investigated the relationship between Apo AIV and CAD reported that Apo AIV levels were significantly lower in the CAD patients compared to the controls (7.6 [SD = 3.5] mg/dL versus 10.4 [SD = 4.1] mg/dL, *P* < 0.001). The inverse associations of Apo AI and Apo IV concentrations with CAD could be explained by the atheroprotective property of these two proteins (32–34). Being a component of high-density lipoprotein (HDL), Apo AI and Apo AIV are involved in the removal of excess cholesterol from the peripheral tissues via the promotion of reverse cholesterol transport (35–36).

Meanwhile, the increase in Apo AI expression in this study could have been due to an antagonistic response to inflammation secondary to recent cardiac muscle injury. A study on Apo E-null mice models has established the anti-inflammatory properties of Apo AI (37). Furthermore, the administration of reconstituted HDL containing plasma-derived Apo AI reduced vascular cell adhesion molecule-1 (VCAM-1) expression following arterial injury (38). However, there is evidence that showed Apo AI conversion from anti-inflammatory particle to pro-inflammatory particle, particularly during an acute-phase response (39–42). Therefore, it could be speculated that enhanced inflammation and/or oxidative stress and the alteration of properties may convert Apo AI to be atherogenic.

Meanwhile, Apo AIV has also been identified to be a positive acute-phase protein in mouse HDL (43). Similarly like Apo AI, the overexpression of Apo AIV in this study could have been in response to the acute inflammatory response in AMI. Another explanation to the contradicted finding in this study and the above-mentioned studies is the difference in the age group of study participants being analysed. The participants in the previous studies were much older with a mean age above 60 years old. Thus, this study might reflect the proteomic changes during AMI in younger populations that are possibly associated with more significant inflammation reaction.

Nevertheless, the non-significant increase in the verification phase might be due to the conflicting roles of Apo AI and Apo AIV in the progression of AMI. The increase was significant during the discovery phase due to the homogeneity in the baseline characteristics and risk factors profiles of both groups. As a result, proteomic changes observed mainly contributed by the inflammation following AMI events. Yet, in the verification phase, the baseline characteristics and risk factors profiles of participants in each group were more varied, heterogeneous and integrated a wider range of cases and controls. This could have given rise to a relatively smaller net increase in the plasma levels of Apo AI and Apo AIV.

Meanwhile, a consistent role of Hp as an inflammatory marker resulted in a significant elevation of plasma concentrations in both phases and thus, could be a potential biomarker for young adults with AMI. This study has also found that Hp was independently associated with young adults with AMI. The positive coefficient indicates that AMI is more likely to occur as the concentration of Hp increases. Other associated risk factors for AMI in young adults were fasting blood glucose, total cholesterol and smoking status. However, a very high OR and wide CI were noted for smoking. This outcome might be due to the extensive variation of data between the groups as the prevalence of smokers in the AMI group was more than double as compared to the prevalence of smokers in the controls. Larger sample size would result in a smaller AOR and narrow CI for smoking variable.

The elevated concentrations of Hp among the AMI patients could have been a regulatory response to the progression of atherosclerosis. Consistently, a large prospective study, Apolipoprotein Mortality Risk Study (AMORIS), has established Hp to be a significant indicator of AMI risk as there was a 4.2-fold risk of AMI in those with high plasma Hp levels (44). In fact, this parameter was almost as predictive of AMI risk as total cholesterol (45). A recent study by Wang et al. (45) reported a strong correlation between Hp levels and Gensini score, which indicated a pro-atherogenic role of Hp in the development of atherosclerosis.

A significant positive correlation between Hp and of hs-CRP reported in this study suggests the potential of Hp as a risk marker for atherosclerosis establishment. This finding was consistent with the finding of Yao et al. (46) who analysed the role of Hp in premature CAD and demonstrated a significant positive correlation between Hp and hs-CRP (*r* = 0.518, *P* < 0.001). Hs-CRP is a well-documented marker of inflammation and potentially a significant player in atherosclerosis, given the inflammatory component of this disease. A significant role of inflammation in young adults with AMI would be mainly attributed to the fact that young AMI is strongly associated with cigarette smoking. Studies have shown that smoking is associated with chronic inflammation and causes a 20%–25% increase in the peripheral blood leukocyte count and increased level of multiple inflammatory markers including hs-CRP and Hp (47–48). Thus, Hp might be useful as an additional biological marker to improve risk stratification of CAD among young adults. Among young adults, conventional risk factors might not adequate to predict CAD. Since young age is considered as a protective factor, therefore, young individuals are more likely to be classified into inappropriate risk levels.

Nevertheless, this study has some limitations. First, smoking pack-year was calculated based on the assumption that the number of cigarettes smoked was consistent over the years. However, smoking pack-year has been recommended as a primary determinant of smoking-related cardiovascular risk as the estimated average number of cigarettes smoked per day and the duration of smoking provides a more complete representation of exposure than the number of cigarettes per day alone (49). Second, the protein spots identified in the discovery phase were restricted to the most abundant plasma proteins due to the limitation of 2-DE method in detecting low protein concentration. However, this approach is still valuable as a screening tool for the discovery of candidate biomarkers. Subsequently, the verification phase confirmed the differential abundance of Hp in human plasma. This study was also limited to the proteomic analysis of young adult with AMI in comparison to the healthy control in the same age group. Therefore, differential proteomic analysis of samples collected from young and elderly patients during the same timeline may result in a better comparison of proteomic profiles between young AMI and the older counterparts.

## Conclusion

In summary, Hp, Apo AI and Apo AIV were significantly up-regulated in young adults with AMI in the discovery phase. However, only plasma concentration of Hp was significantly higher in young AMI patients relative to the healthy controls in the verification phase. Hp was also independently associated with AMI in young adults and positively correlated with hs-CRP. Therefore, Hp is a potential biomarker of AMI in young adults.

## Figures and Tables

**Table 1:** Baseline characteristics of all subjects in the discovery and verification phases

	Discovery phase			Verification phase		
	
	AMI (*n* = 10) mean (SD)	Control (*n* = 10) mean (SD)	*P*-value	AMI (*n* = 40) mean (SD)	Control (*n* = 80) mean (SD)	*P*-value
Age, years	39.7 (3.7)	38.2 (3.7)	0.380	37.8 (5.5)	33.0 (6.2)	< 0.001
BMI, kg/m^2^	25.33 (3.36)	24.99 (5.31)	0.712	29.62 (6.88)	26.26 (4.70)	0.002
Current smoker, *n* (%)	10 (100%)	10 (100%)	-	33 (82.5%)	27 (33.8%)	< 0.001
Smoking pack, years*	20.0 (9.5)	4.8 (8.7)	0.003	16.0 (13.8)	4.8 (3.8)	< 0.001
SBP, mmHg	127.8 (19.4)	114.2 (3.2)	0.056	134.9 (19.6)	121.6 (10.1)	< 0.001
DBP, mmHg	84.1 (13.0)	71.6 (4.4)	0.010	87.7 (14.8)	78.5 (7.5)	< 0.001
Fasting glucose, mmol/L*	6.19 (0.48)	5.48 (0.47)	<0.001	6.30 (1.11)	4.90 (0.50)	< 0.001
Total cholesterol, mmol/L	5.33 (1.61)	6.25 (1.10)	0.146	5.63 (1.56)	5.95 (1.09)	0.235

Notes: Values are presented as means (SD);

* median (IQR) or numbers; (%); data was analysed using independent Student’s *t*-test for normally distributed data;

* Mann Whitney U test for not normally distributed data and Chi squared test for categorical data;

BMI = body mass index; SBP = systolic blood pressure; DBP = diastolic blood pressure; AMI = acute myocardial infarction

**Table 2:** Proteins that were differently expressed in AMI patients relative to controls as identified by MALDI-TOF mass spectrometry

Spot	Protein name	Account number	Nominal mass	Calculated pI	Sequence coverage	Fragment
SSP 7201	Hp	P00738	45177	6.13	8%	LRTEGDGVYTLNNEK
SSP 2501	Apo A1	P012647	30759	5.56	48%	DLATVYVDVLK
SSP 1601	Apo A-IV	NP_000473.2	35344	5.28	31%	KLVPFATELHER

Note: MALDI-TOF = matrix-assisted laser desorption/ionisation of time of flight; pI = isoelectric point

**Table 3:** Association between Hp and AMI in young adults

Variables	B	Crude OR	AOR	95% CI		*P*-value
Age	0.13	1.14	1.14	1.00	1.30	0.050
BMI	0.05	1.11	1.06	0.90	1.24	0.457
Smoking(1)	4.70	14.14	110.48	7.54	1617.93	0.001
Cholesterol	−0.78	0.75	0.45	0.24	0.86	0.015
Fasting glucose	0.71	2.94	2.03	1.22	3.58	0.013
SBP	0.06	1.06	1.07	0.99	1.15	0.066
DBP	0.02	1.08	1.02	0.92	1.12	0.683
Hp	0.01	1.01	1.01	1.00	1.03	0.025

Notes: multivariable binary logistic regression analysis; BMI = body mass index; SBP = systolic blood pressure; DBP = diastolic blood pressure; SE = standard error; OR = odds ratio; CI = confidence interval; (1) compared to non-smoker
